# The Genetic Background of Ankylosing Spondylitis Reveals a Distinct Overlap with Autoimmune Diseases: A Systematic Review

**DOI:** 10.3390/jcm14113677

**Published:** 2025-05-23

**Authors:** Theodora Zormpa, Trias Thireou, Apostolos Beloukas, Dimitrios Chaniotis, Rebecca Golfinopoulou, Dimitrios Vlachakis, Elias Eliopoulos, Louis Papageorgiou

**Affiliations:** 1 Laboratory of Genetics, Department of Biotechnology, Agricultural University of Athens, 11855 Athens, Greece; theozorb@med.uoa.gr (T.Z.); thireou@aua.gr (T.T.); r.golfinopoulou@aua.gr (R.G.); dimvl@aua.gr (D.V.); 2 Department of Biomedical Sciences, University of West Attica, Agioy Spyridonos, 12243 Egaleo, Greece; abeloukas@uniwa.gr (A.B.); dchaniotis@uniwa.gr (D.C.)

**Keywords:** genomics, Ankylosing Spondylitis, rheumatoid arthritis, psoriasis, autoimmune diseases, disease genomic grammar, single-nucleotide polymorphisms

## Abstract

**Background**: Ankylosing Spondylitis (AS) is a rare autoinflammatory disorder affecting 0.1–1.4% of the population, with increasing recognition over the past 20 years. Although the specific causes of AS remain unclear, the presence of the *HLA-B27* gene is associated with increased risk, though only 1–5% of carriers develop the disease. Despite extensive research, no definitive lab tests exist, and many patients are diagnosed years after symptom onset. **Methods**: In the present study, in order to investigate the disease’s genetic background in correlation with autoimmune diseases, a metanalysis has been performed following PRISMA guidelines using Scopus and PubMed publications towards extracting single-nucleotide polymorphisms (SNPs) of high importance for the disease. Moreover, the polymorphisms have been annotated and analyzed using information from several databases, including PubMed, LitVar2, ClinVar, and Gene Ontology. **Results**: From 1940 screened titles and abstracts, 57,909 studies were selected, with 539 meeting the inclusion criteria. The genetic background of AS is described through 794 genetic variants, of which 76 SNPs are directly associated with AS (Classes A and B), predominantly located in intronic regions. *ERAP1* and *IL23R* emerged as key genes implicated in AS, while chromosomes 1, 2, and 5 accumulated the most associated SNPs. Functional enrichment revealed strong associations with immune regulation and interleukin signaling pathways, particularly *IL6* and *IL10* signaling. *IL-6* promotes inflammation in AS, while *IL-10* tries to suppress it, acting as an anti-inflammatory cytokine. Of the 78 AS-related SNPs, 16 were unique to AS, while 66 were common to autoimmune diseases, especially rheumatoid arthritis (RA) and psoriasis (PsO), suggesting genetic overlap between these diseases. **Conclusions**: This study creates a comprehensive genetic map of AS-associated SNPs, highlighting key pathways and genetic overlap with autoimmune diseases. These findings contribute to understanding disease mechanisms and could guide therapeutic interventions, advancing precision medicine in AS management.

## 1. Introduction

Ankylosing Spondylitis (AS), a subtype of axial spondyloarthritis (axSpA), is an autoinflammatory disorder and it belongs to a group of disorders called seronegative spondyloarthropathies (SpAs), which are characterized by the absence of specific autoantibodies. This group includes AS, psoriatic arthritis (PsA), inflammatory bowel disease (IBD)-associated arthritis, reactive arthritis (ReA), and undifferentiated SpAs [1], and it affects approximately 0.1% to 1.4% of the population in Europe, with men being more commonly diagnosed than women, at a ratio of about 2:1. Clinical symptoms typically begin before the age of 45 and primarily affect the axial skeleton, causing inflammatory back pain and progressive spinal stiffness due to sacroiliac joint inflammation and pathological bone remodeling. This can lead to vertebral fusion, reduced mobility, and impaired quality of life [2,3]. Beyond axial involvement, AS may present with peripheral arthritis, enthesitis (commonly at the heels), and extra-articular features, such as anterior uveitis, psoriasis, and IBD, reflecting shared pathogenic mechanisms [4]. These features suggest that AS is not an isolated condition, but rather part of a broader spectrum of immune-mediated disorders.

Autoimmune and autoinflammatory diseases, including AS, pose significant challenges in terms of early diagnosis and targeted treatment. One of the primary difficulties lies in the heterogeneous nature of these diseases, where symptoms may be subtle or nonspecific in the early stages, often overlapping with other inflammatory or musculoskeletal conditions. In the case of AS, assessment is mainly achieved through clinical examination in combination with an X-ray/magnetic resonance imaging (MRI) in the pelvic area. However, symptoms may not appear in the early stages of the disease or may not lead to an accurate diagnosis [5]. Alternatively, in cases where there are no radiological findings—described as non-radiographic axSpA (nr-axSpA)—clinical examination combined with the finding of the histocompatibility antigen *HLA-B27*, which is the main genetic marker for this disease (as well as all SpAs) with an odds ratio of, approximately, 60%, can lead to a diagnosis; however, only 1–5% of those who carry the allele develop AS, meaning that its predictive value remains limited and highlights the need for more precise genetic and biomarker-based tools [6].

Early detection and prompt treatment are crucial to ensure a high quality of life for patients. The treatment of AS has evolved significantly in recent years, but challenges remain in tailoring therapies to individual patients. Traditional approaches primarily focus on managing symptoms and slowing disease progression. Nonsteroidal anti-inflammatory drugs (NSAIDs) are the first line of treatment, offering relief from pain and stiffness, and may be complemented by non-steroidal anti-inflammatory drugs (NSAIDs), to reduce inflammation when peripheral arthritis is present [2,7,8]. However, when these are not sufficient, biologic agents are recommended, mainly tumor necrosis factor (*TNF*) inhibitors, such as etanercept, infliximab, and adalimumab, which have become a cornerstone in the management of AS, effectively targeting the inflammatory processes that drive the disease. Also, new approaches include interleukin-17 (*IL-17*) inhibitors, like secukinumab and ixekizumab, which have shown promising results in reducing disease activity and, even more recently, *JAK* inhibitors [9]. While current therapies have shown specificity in targeting key inflammatory pathways, they are not universally effective, and treatment responses can vary widely among patients. This variability highlights the complex and heterogeneous nature of AS and underscores the need for a deeper understanding of its underlying immunogenetic mechanisms.

### 1.1. Pathogenesis of AS—Immunological Insights

The immunopathogenesis of AS involves both innate and adaptive immune responses triggered by mechanical stress and microinjuries in the axial skeleton. Key cytokines such as *TNF-α*, *IL-17*, and *IL-23* promote chronic inflammation and abnormal bone remodeling, whereas *IL-23* stimulates *IL-17*–producing cells, including Th17, γδ T cells, MAIT cells, and ILC3s, which drive tissue inflammation [10,11,12]. These cells are particularly responsive to *IL-23* and play a crucial role in the early initiation of inflammation. Complementarily, the adaptive immune system sustains chronic inflammation through the Th17 immunological response, driven by *IL-23*, which produces *IL-17* and *IL-22*, perpetuating inflammation and contributing to pathological bone remodeling. Dysregulation of the *IL-23*/*IL-17* axis plays a central role in driving chronic inflammation and tissue damage. Although the exact triggers remain unclear, microbial dysbiosis and gut inflammation are suspected to initiate aberrant immune activation in genetically predisposed individuals [13,14].

Genetic factors such as *HLA-B27* and *ERAP1* variants influence immune cell function and cytokine profiles, by shaping the immunopeptidome (the repertoire of peptides bound and presented by the MHC-class molecules). The main hypothesis regarding the *HLA-B27* susceptibility mechanism suggests that this allele misfolds, possibly due to a change at Cys 67 that does not allow the formation of a disulfide bond and consequently causes ER stress, which activates the UPR mechanism [10,11,15,16]. Additionally, it has been proposed that due to molecular mimicry between HLA-B27 (amino acids 72–77) and the bacterial nitrogenase of *Klebsiella pneumoniae* (amino acids 188–193), this variant may confer risk to AS after an individual becomes infected by this bacterium [17].

### 1.2. Genetic Background

**Figure 1:**
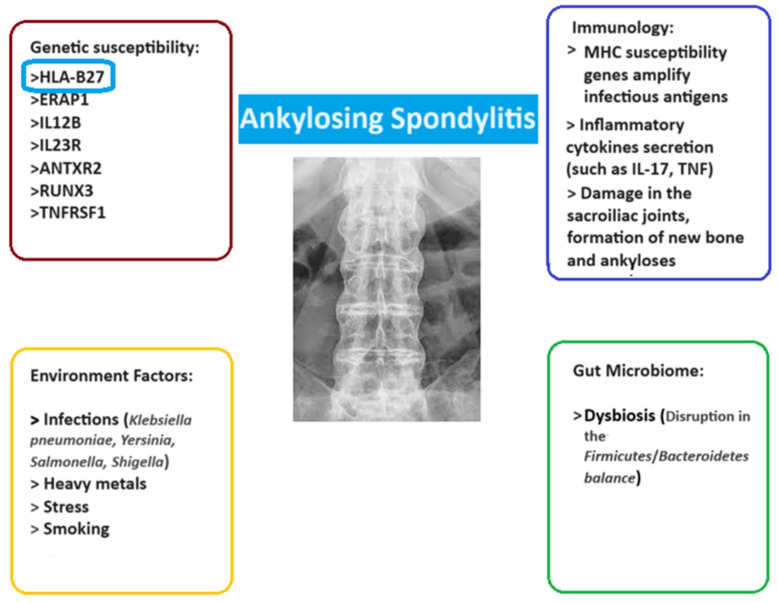
Previously reported factors that play a key role in AS pathogenesis.

Although inflammation in AS is driven by immune dysregulation and cytokine cascades, these processes are orchestrated by a complex genetic background. AS is a polygenic disease, with more than 100 genetic associations. The *HLA-B27* variants in the major histocompatibility complex (MHC) play a key role in the disease manifestation (Figure 1). Over 80% of patients with spondyloarthritis have this variant, yet only 1–5% of people who carry the allele develop the disease, making it a complex disease about which we still have limited knowledge [18,19]. One study revealed that sub-alleles HLA-B*27:02, 04, and 05 were identified in 87.5% of patients but were absent in controls [20]. In this study, there was a strong association with a family history of back pain and elevated blood biomarkers (CRP, ESR), while no significant association with age, gender, or environmental factors was found. In addition, a confirmed association with the disease has been recorded for 12 different genetic loci in Europeans, namely *ANTXR2*, *CARD9*, *ERAP1*, *IL12B*, *IL23R*, *KIF21B*, *PTGER4*, *RUNX3*, *TBKBP1*, *TNFRSF1A*, and chromosomes 2p15 and 21q22, which are all related to immune proteins (Figure 1) [21]. Variations in these loci may altogether affect the disease; however, the amount of risk imposed by each variation, separately, is too small to be a criterion—by itself—in the diagnosis of the disease [19,22,23]. A comparison of genetic models of AS was conducted by a study to assess a possible mode of inheritance, resulting in different recurrence risk depending on the degree of relation, with monozygotic twins having a 63% recurrence, first-degree relatives an 8.2% risk, and dropping to 0.7% for third-degree relatives, revealing, at the same time, no significant difference between parent–child and sibling recurrence risk [24]. In a European nested case–control study the overall familial risk, defined as the odds ratio, was 19.4 (95% CI 18.1, 20.8), with similarities between different relative types and sex, and it was much higher in cases with more than one AS-affected relatives (odds ratio of 68.0 (95% CI 51.3, 90.1)) [25].

### 1.3. Complex Traits and Autoimmunity

Like AS, many autoinflammatory and autoimmune diseases are polygenic, meaning a set of genetic factors contribute to the disease together [19,21]. Many of these have been identified by using genome-wide association studies (GWASs) of a group of patients compared to the human reference genome. These studies indicate that many autoimmune diseases share some common risk factors, suggesting that there is a genetic basis for autoimmunity [26,27,28,29]. More specifically, *HLA*, *PTPN2*, *ANTXR2*, *TYK2*, and *IL2RA* are some of the genes that were found to have an association with several autoimmune and autoinflammatory diseases, including AS, ulcerative colitis (UC), Crohn’s disease (CD), and psoriasis (PsO) [30,31]. Further knowledge of these relationships can help in gaining a deeper understanding of the underlying molecular mechanisms [32].

Quite often, this is not an easy task since most of the associated genetic variants are placed in non-coding areas of the genome and act as regulators of other genes. Also, it is worth noting that GWASs cannot fully explain the estimated heritability. More specifically, in AS, *HLA-B27* can explain only 20% of AS heritability, while the other 12, non-MHC loci which have been confirmed to be AS-associated in Europeans, are estimated to explain another 10% of AS heritability, meaning only 30% of total AS heritability can be explained at the moment by these genetic factors [33]. Therefore, this raises questions about possible epigenetic factors that additionally contribute to the onset of the disease and remain yet unknown.

It has been proposed that environmental factors, including infections (primarily *K. pneumonia* but also *Salmonela* spp., *Yersinia* spp., and *Shigella* spp.), heavy metals, stress as well as the gut, may also play a role in the pathogenesis of AS—mainly through gut dysbiosis, which disrupts the barrier to exogenous bacteria or viruses and has also been studied in the case of autoimmune diseases such as IBD [17,22,34]. Moreover, epigenetic modifications—including DNA methylation, histone alterations, and non-coding RNAs like microRNAs—can influence gene expression without altering the underlying DNA sequence. These changes are responsive to environmental stimuli, including infections, smoking, and gut microbiota imbalances, which are known to affect AS development. For instance, aberrant DNA methylation patterns have been observed in genes associated with immune regulation and inflammation in AS patients [34,35]. Additionally, specific microRNAs have been implicated in modulating inflammatory pathways relevant to AS, and previous studies have reported that polymorphisms in microRNAs on X and other chromosomes, such as miR-146a, miR-155, miR-125a-5p, miR-151a-3p, miR-22-3p, and miR-199a-5p, could be involved in the different clinical presentations of SpAs as well as disease activity, acting either as regulators of epigenetic enzymes or as targets of epigenetic silencing [36].

The genetic basis of autoimmunity is strongly anchored in the major histocompatibility complex (MHC), particularly the *HLA* region, which remains the most significant and consistently replicated risk factor across multiple autoimmune diseases. Specific *HLA* alleles include *HLA-DR3* and *HLA-DR4* (associated with type 1 diabetes—T1D), as well as *HLA-DRB1* (associated with rheumatoid arthritis—RA and multiple sclerosis—MS), *HLA-DQB1* (associated with MS), and *HLA-B27* (associated with AS) [37,38,39]. Beyond the MHC, numerous non-HLA genes also contribute to autoimmunity by modulating immune signaling and tolerance. Key examples include STAT4, which influences Th1 and Th17 responses and has been implicated in IBD, RA, and other conditions; ERAP1, involved in antigen processing, which has been implicated in AS and PsO; IL12R and IL23R, which affect cytokine signaling pathways crucial in T cell differentiation and have been implicated in AS, IBD, and PsO; *TYK2*, a mediator of *JAK-STAT* signaling that has been implicated in AS, IBD, PsO, RA, MS, and T1D; *PTPN22*, a phosphatase involved in *TCR* signaling that has been implicated in RA, T1D, and SLE; and also *CTLA4*, a critical regulator of T cell activation that has been implicated in T1D and other conditions [40]. Together, these loci highlight the polygenic and complex nature of autoimmune susceptibility, driven by both antigen presentation and immune regulatory networks.

These findings clearly indicate that genetic variations do not act in isolation but rather influence immune cell behavior, cytokine profiles, and tissue-specific responses. By combining genomic data with clinical features—such as symptom onset, radiographic findings, and treatment outcomes—clinical genomics could bridge the gap between genotype and phenotype. Ultimately, the application of clinical genomics, particularly when combined with tools like immunophenotyping and longitudinal patient monitoring, is key to uncovering shared and unique mechanisms in autoimmunity. This approach also enables the identification of subtypes within AS, contributing to a more individualized model of care.

### 1.4. Aim of the Study

Given the complexity and heterogeneity of AS, there is a pressing need to integrate genetic and immunological insights with clinical practice. Therefore, in this systematic review, the possibility of the existence of a representative number of polymorphisms related to AS through scientific publications was examined, as well as how many of them appear to be associated with autoimmune diseases. The aim is to evaluate genetic variants that play a central role in the predisposition to and development of AS. This knowledge can act as beneficial knowledge in personalized medicine by using a genotype approach to define different AS subtypes, as well as to identify distinct risk factors and specific molecular pathways, leading to an early-stage diagnosis and targeted therapies that maximize efficacy and minimize risk of toxicity. Moreover, as an outcome, it can highlight the genetic overlap between different autoimmune diseases, assisting research regarding comorbidity and the underlying molecular mechanisms involved in autoimmunity. These results can also be combined with whole-exome sequencing (WES) and whole-genome sequencing (WGS) data from candidate patients in order to fully capture the complexity of disease development and lay the foundations for the estimation of genetic risk scores based on the genetic background and ethnicity of patients.

## 2. Materials and Methods

**Figure 2:**
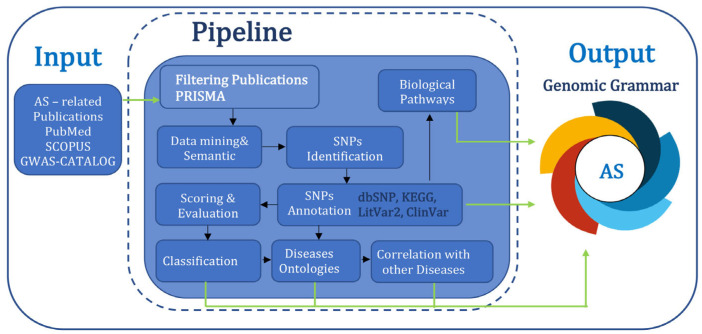
Overview of the bioinformatics pipeline utilized in this study.

This study is based on a specific bioinformatic pipeline with separate processes, in order to estimate the disease genomic grammar (DGG) of AS [41] (Figure 2).

### 2.1. Dataset Collection and Pre-Analysis

As in previous studies, the data presented in this paper were obtained from publications written in English and included in the PubMed and SCOPUS databases from 1940 to March 2025 [42,43,44]. Research was performed based on the key term “Ankylosing Spondylitis” and related keywords, including “axial Spondyloarthritis” and “axSpA”. Consequently, selected publications have been included in the dataset from the GWAS Catalog database after searching for the same terms [42]. A filtering process was performed by using regular expressions in MATLAB 2020a, and duplicated publications or reports, as well as publications or scientific reports (a) without a title and abstract, (b) without any direct correlation with AS, and (c) without any SNP identifier that corresponds to the dbSNP database, were removed from the dataset. The detailed inclusion and exclusion criteria and numbers are shown in the PRISMA flowchart (Supplementary Figure S1). Afterwards, a pre-analysis was performed by using regular expressions towards screening the publications and scientific reports that were contained in the filtered dataset, in addition to identifying and collecting the SNP identifiers. As a result, a new dataset was created, which summarizes all of the unique SNPs found to be associated with AS, as well as the number of different publications and scientific reports described along with their characteristic identifiers according to the biological database of origin (PubMed or SCOPUS).

### 2.2. Data Annotation

The SNPs collected after pre-analysis were re-evaluated to identify the ones that are directly related to AS and to confirm the information extracted. All findings were assessed for their accuracy by performing separate searches in the dbSNP database. Those that were found to be different based on existing information were identified and adjusted based on the information provided by the dbSNP database, and all unrelated identifiers were eliminated. Moreover, they have been annotated with information from relevant databases, such as dbSNP [45], a database containing detailed SNP information such as variation type, genetic locus, and clinical differences, and ClinVar [46], offering insights into phenotypes that have been observed in patients carrying these variants, through clinical experiments as well as information regarding disease ontologies. Additionally, LitVar2 [47], which uses semantic tools and summarizes information regarding a variant through Pubmed publications, was employed, as were the OMIM [48] and KEGG Pathway [49] databases to identify related genes, biological pathways, and chemical compounds. Lastly, GO enrichment was performed with DAVID tools [50] and cross-validated with Reactome [51].

### 2.3. Data Classification and Evaluation

As in previous studies [43,44], each SNP was classified according to the scoring function described below, and the outcome was manually evaluated using the available literature results in order to avoid a negative correlation of an SNP with AS or an autoimmune disease. Several criteria have been manually examined, and the available publications or scientific reports have been separated into (a) human studies, (b) GWASs, meta-analyses, or any type of original SNP population analysis, and (c) presentation of SNP association in the abstract of a scientific report. Polymorphism connections with an unclear correlation with AS or autoimmune diseases were removed from the final dataset.

**Score =** (VNorFrePub ∗ 0.1) + (VNorFreLitVar2 ∗ 0.4) + (VClinVar ∗ 0.2) + (VNorRelDis) ∗ 0.3) (1)

where VNorFrePub represents the normalized frequency of the identified SNPs from the publication dataset (Max = 1/Min = 0); VNorFreLitVar2 represents the normalized frequency of the identified SNPs based on the LitVar2 database output and AS connections (Scalar value, Max = 1/Min = 0); VClinVar represents the normalized frequency of the identified SNPs based on the ClinVar database (Scalar value, Max = 1/Min = 0), and correlations with autoimmune diseases; and VNorRelDis represents the normalized frequency of SNPs found in relative diseases, namely RA, PsO, and MS based on the LitVar2 Database (Scalar value, Max = 1/Min = 0). Lastly, the SNP dataset was classified using the following scoring function:

$$\begin{array}{l} \mathrm{Scoring}\;\mathrm{Function}\\ \mathrm{Strong-associated}\;\mathrm{SNPs/Class}\;A:\;\mathrm{score}\;\geq \;0.6.\\ \mathrm{High-associated}\;\mathrm{SNPs/Class}\;B:\;0.6>\mathrm{score}\;\geq \;0.4.\\ \mathrm{Associated}\;\mathrm{SNPs/Class}\;C:\;0.4>\;\mathrm{score}. \end{array}$$

The synthesis and presentation of the results were based on the extracted and evaluated information. The highly associated polymorphisms (Classes A and B) were analyzed based on (a) the type of polymorphism, (b) the reference gene, (c) the reference chromosome, and (d) the correlated diseases. Additionally, they were separated into the unique polymorphisms that are found to be involved in AS and the polymorphisms that are found in autoimmune diseases using Venn diagrams and the available literature.

## 3. Results

### 3.1. The Genomic Grammar of Ankylosing Spondylitis

**Figure 3:**
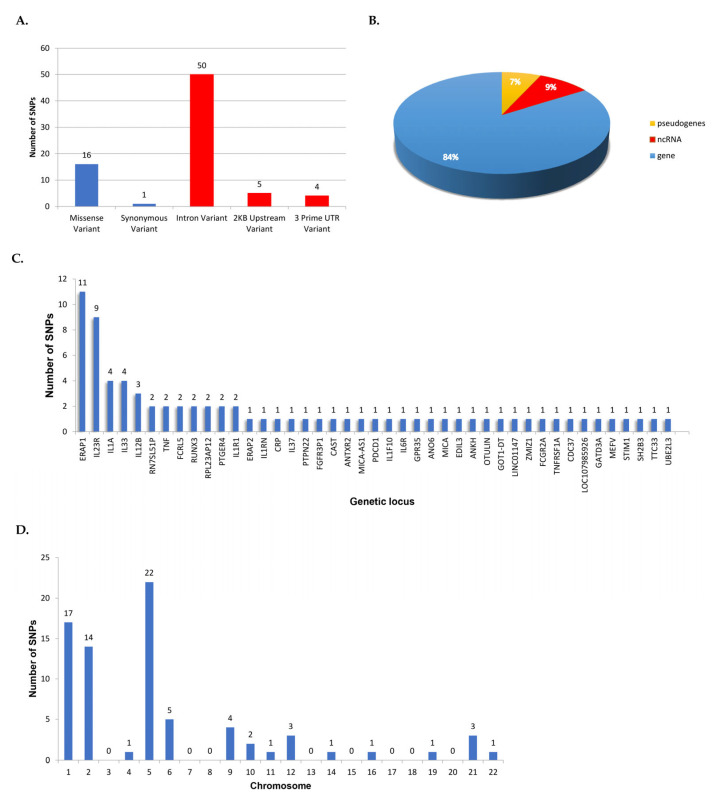
Statistical analysis of the AS-related SNPs that were classified in Classes A and B. (**A**) Categories of SNPs. (**B**) Characteristic regions in the genome. (**C**) Number of SNPs per genomic characteristic region. (**D**) Distribution of SNPs per chromosome.

A total number of 57,909 publications containing or associated with the term “Ankylosing Spondylitis’” were retrieved from PubMed and SCOPUS, including information dated between 1940 and 2025. Most of them were registered in the last 20 years, indicating an increased awareness from the scientific community. After a filtering process based on the PRISMA flowchart, 539 publications were selected (Supplementary Table S1), from which 793 SNPs have been identified. The dataset created was then annotated, evaluated, and classified based on the information found in other biological databases such as NCBI dbSNP, LitVar2, ClinVar, and KEGG Pathway. The final dataset, corresponding to the disease genomic grammar of AS, contains 793 genetic variants, of which 32 SNPs are strongly associated (Class A) with AS tand 44 are highly associated (Class B), while a vast majority, 717 of them, received a lower score (Class C), suggesting either a weak association or that more research is needed to assess their importance (Table 1, Supplementary Table S2). For the purposes of this study, further analysis focuses on SNPs of Classes A and B (Supplementary Table S2). The number of significant SNPs rises to a total of 76 and affects 43 different genetic loci, from which, 376 are genes, 3 are pseudogenes, and 4 are ncRNA (Figure 3). Most of these variants lie on non-coding regions, acting as regulators of genes, 50 of which are intronic; four lie on three prime UTR regions; and five are 2KB upstream variants (Figure 3). A smaller portion of variants lie on coding regions, 16 of which cause a missense mutation, ultimately altering the protein’s ability to interact, and 1 is synonymous, altering gene expression levels (Figure 3).

Most of the SNPs reported affect ERAP1, an aminopeptidase involved in trimming HLA class I-binding precursors, which has been widely studied as a risk factor for AS. This gene’s variants are thought to confer risk, acting through an epistatic interaction with *HLA-B27* as well as other MHC proteins [52]. Additionally, a great number of SNPs were found to affect IL23R, a receptor involved in *IL23A* signaling that is known to be related to AS as well as some autoimmune diseases, and has been previously studied as a possible therapeutic target (Figure 3) [22,53]. Also, at the chromosome level, chromosomes 1, 2, and 5 accumulated the most SNPs (Figure 3). Interestingly, although most of the SNPs (84%) affected genes, 6.8% affected pseudogenes, and 9% affected ncRNAs. It is important to note that recent studies suggest that SNPs in intronic regions and pseudogenes, though their functions remain poorly understood, may influence gene regulation through mechanisms such as alternative splicing and enhancer activity [54]. This could possibly explain a part of the ‘missing’ AS heritability; however, it is good to keep in mind that while GWASs can identify genetic associations, functional validation is essential to establish causal relationships, and that there are some inherent limitations of GWASs in multifactorial diseases. Further analysis of protein interactions and related pathways showed a strong association between the involved genes and immunological pathways, especially cytokine and interleukin signaling, in which 12 genes were included, namely *IL6R*, *IL33*, *IL12B*, *IL37*, *IL1R1*, *IL1A*, *TNFRSF1A*, *TNF*, *IL23R*, *IL1RN*, *IL1F10*, *LTBR*, and *SH2B3*. The involvement of interleukins in signaling in AS is well studied, especially for the *IL-23*/*IL-17* axis, which plays a role in the Th17 immune response and has been previously associated with many immune-mediated inflammatory diseases, including RA, PsO, IBD, and MS. It has been shown that exposure of Th17 cells to *IL-23* induces pathogenesis and leads to the expression of their master regulator, *RORγt*, as well as to the production of pro-inflammatory cytokines [55]. Also, the *IL-23/IL-17* axis has attracted attention as a potential therapeutic target for AS. While NSAIDs, medicines which target the cyclooxygenase (COX) enzymes, relieving pain and reducing inflammation, are considered as the first-line treatment for AS, there are cases in which they might not be successful in suppressing AS symptoms, or side effects may occur due to their long-term use. In these cases, treatment with biologics, such as the inhibitors anti-TNF and anti-IL17, seems very promising for AS patients [2,10,55].

Besides immunological pathways, the analysis also showed association with pathways that involve signal transduction, metabolism, programmed cell death, the transport of small molecules, and gene expression. Furthermore, gene ontology analysis showed that the encoded proteins are involved in biological processes that pertain to the immune system, such as cytokine signaling, the inflammatory response, the positive regulation of IFN-γ production, the positive regulation of *JAK-STAT*, and the cellular response to LPS. It also showed that most of the proteins are located at the external side of the plasma membrane, as well as in the extracellular space and at receptor complexes, while their molecular functions involved cytokine binding, especially of *IL-1*, and metallo-aminopeptidase activity.

Similar findings have been previously reported in studies that investigate the possible role of the immune system and bacterial factors in AS pathogenesis. It has been suggested that *HLA-B27*-positive individuals are more susceptible to AS after microbial infection (especially by *K. pneumoniae*), which leads to increased LPS levels and, therefore, triggers the activation of innate immune cells. These, in response, promote inflammation, a process driven by cytokine production (including *TNF*, *IL-23*, and *IL-17*), while hematopoietic stem cells invoke *NF-κB*, *RANKL*, and *M-CSF*, leading to abnormal bone formation [56]. In addition, it has been previously suggested that the *IL-1* gene cluster plays a role in AS, due to the observation that there is an aberrant expression of this cytokine in AS patients (among others, including *TNF* and *IL-17*), and this cytokine has also been used as a potential therapeutic target; however, the causal relationship between this gene and AS pathogenesis needs further investigation [57]. Also, metalloaminopeptidase activity has been previously studied in association with AS through the role of endoplasmic reticulum aminopeptidase 1 (*ERAP1*) and *ERAP2* enzymes, which work closely with MHC-I molecules (such as the *HLA-B27*) for antigen presentation [52].

Furthermore, these SNPs were examined for possible overlapping associations with autoimmune diseases, and it was found that many of the AS-related genetic variants are also associated, primarily, with PsO as well as RA and secondly with IMD, such as CD and UC (Table 2). All of these diseases have been previously studied in association with AS; however, cases reported for comorbidity are quite rare due to the rarity of AS patients alone [58,59,60]. In such cases, where clinical reports are scarce due to disease rarity or a difficulty in diagnosing patients, genetic research can prove to be very useful for detecting possible associations. However, these associations do not prove causality and should be carefully examined [61].

Overall, many of the AS-related SNPs were found to be associated with autoimmune diseases; however, 16 SNPs were found to be uniquely associated with AS, with only a few studies trying to associate them with other diseases (Supplementary Figures S2 and S3). These are the rs7711564 and rs27038 located at *ERAP1*, the rs6692977 located at *FCRL5*, the rs27037 located at the *CAST* gene, the rs4333130 located at *ANTXR2*, the rs2242944 located at RPL23AP12, the rs116488202 located between *MICA*, the rs26307 located at *OTULIN*, the rs4648889 located at *RUNX3*, the rs1894399 and rs2856836 located at *IL1A*, the rs2192752 located at *IL1R1*, the rs27356 located at *ANKH*, the rs3750996 located at *STIM1*,the rs4349859 located at *MICA-AS1*, and, lastly, the rs1929992 located at *IL33* and LOC107987046. Especially in the case of rs116488202, this SNP has also been greatly associated with acute anterior uveitis, a very common extra-articular manifestation of AS, which has been noticed in 25,8% of AS cases [62].

**Figure 4:**
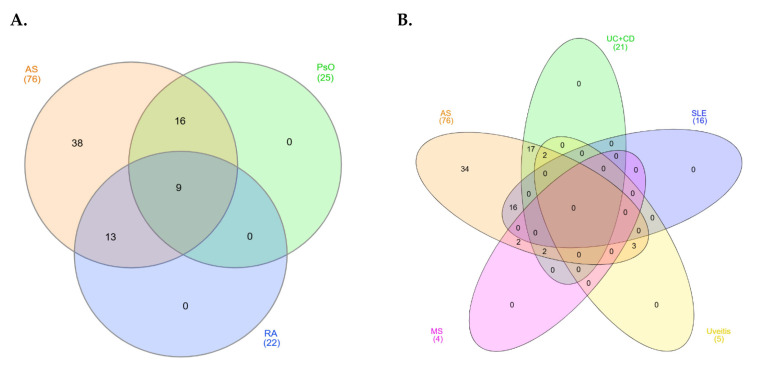
Venn diagrams. (**A**) Similarities between AS-associated SNPs (Classes A and B) and genetic overlap with RA and PsO. (**B**) Similarities between AS-associated SNPs (Classes A and B) and genetic overlap with IBD, SLE, uveitis, and MS. For IBD, we took the total unique SNPs found between UC and CD.

In this study, we chose to focus on the genetic variants that AS shares with RA and PsO, two chronic autoimmune diseases that have more established genetic overlap and are more commonly compared in the literature. These have been previously strongly associated with MHC molecules (*HLA-DRB1* shared epitope alleles and HLA-C*0602, *respectively)*, but altogether hold some etiological and clinical differences (Figure 4) [63,64]. Moreover, we examined the genetic overlap between AS and autoimmune diseases that are more distantly related or less genetically overlapping with AS. These include IBD, systemic lupus erythematosus (SLE), uveitis, and MS, and the SNPs that appeared in at least three of these diseases were further discussed (Figure 4). The aim was to investigate possible underlying molecular mechanisms that these autoimmune diseases may share with AS. Therefore, the shared genetic variants were collected and analyzed regarding their molecular mechanisms as well as the gene ontology and the related pathways of the encoded proteins that they affected (Figure 3).

### 3.2. Genetic Overlap Between AS and Stongly Associated Autoimmune Diseases—RA and PsO

In this study, nine SNPs were found to be common in the diseases AS, RA, and PsO, located in six distinct genetic loci, namely *IL23R*, *IL6R*, *CDC37*, *IL12B*, *TNF*, and *PTPN22*, most of which pertain to immunological processes (Figure 4, Table 3). More specifically, *IL23R* binds *IL-23*, promoting Th17 cell differentiation; IL6R binds IL-6 and initiates inflammatory responses through *JAK*/*STAT3* signaling; *CDC37* acts as a co-chaperone that stabilizes protein kinases via *HSP90*, including immune-related kinases, and indirectly can affect signaling pathways through kinase stabilization (*JAKs*, *IKKs*); *IL12B* encodes a p40 subunit shared by *IL-12* and *IL-23*, which activate Th1 and Th17 cells, respectively; *TNF* is a pro-inflammatory cytokine that promotes immune cell activation, apoptosis, and inflammation; and, lastly, *PTPN22* negatively regulates T cell receptor (TCR) signaling.

Within the locus of *IL23R*, four SNPs (rs11209026, rs2201841, rs1343151, and rs10489629) were found to be associated with the three diseases, with one being exonic (rs11209026) and causing a missense variation, while the other three were intronic. More specifically, the missense SNP leads to an Arg381Gln mutation that affects the cytoplasmic tail of the receptor of *IL23*. This domain is responsible for signal transduction by interacting with its associated *JAK2* kinase; therefore, this missense SNP may functionally affect the *IL-23R* transducing pathway [65]. It has also been shown that this SNP reduces *IL-17A* and *IL-22* serum levels [66]. In some cases, protective haplotypes, including R381Q, may influence mRNA splicing by modifying the SF2 splicing enhancer binding site, leading to increased production of a soluble *IL-23R* isoform (*IL23RΔ9*), which binds *IL-23* and blocks its pro-inflammatory signaling pathway. Thus, a therapeutic strategy using antisense oligonucleotides to induce this splicing shift offers a promising approach with which to reduce *IL-23* signaling in autoimmune conditions [67]. Interestingly, while it has been reported that this SNP is protective for AS, in a meta-analysis it has been suggested that it confers risk to RA and it has also been associated with PsO through GWASs [68,69]. On the other hand, the intronic rs2201841 was found to be associated with AS susceptibility, and the G allele was also associated with an increased risk for PsO, as shown in GWASs [70,71,72,73]. However, the latter has been shown to be protective in the case of RA, as shown in a meta-analysis [69]. In the case of the intronic SNPs (rs1343151 and rs10489629), the A and C allele, respectively, seemed to confer risk to both AS and PsO, as shown in GWASs [70,71,72,73]. Lastly, in the case of RA, these SNPs were associated with increased risk, and even though previous studies showed controversial results, another study suggests that the susceptibility character of these SNPs might be explained by studying the surrounding haplotype, rather than the SNPs alone [74].

Another SNP, rs4129267, located at intron 8 of *IL6R*, might regulate gene expression, as it has been shown that patients who carried the T allele in homozygosity had 73% higher concentrations of serum *IL6R* [22]. It could also predict a worse ASAS-20 (Assessment of Spondyloarthritis international Society) response in a Taiwanese population. This SNP has been associated with both AS and RA susceptibility [75,76], but was shown to be associated with reduced PsO activity in GWASs [77]. Also, the intronic rs35164067 located in *CDC37* has been associated with AS susceptibility in GWASs and has also been used in an RA association study, investigating a possible connection between SNPs and anti-citrullinated protein antibody (ACPA)-positive RA genetic risk score [22,78]. On the contrary, it was found to be protective of PsO [22,79]. rs3212227, located at the 3′ UTR of the *IL12B* region, has been suggested to be protective against AS [80]. However, another study showed that this allele may amplify the genetic risk for AS, as well as maybe contributing to elevated *IL-23* and *IL-12p40* serum levels if individuals that carry it also carry the rs17860508 allele 2, suggesting a leading role for rs17860508 in genetic susceptibility imposed by *IL12B* polymorphisms [81]. Although its relationship with AS needs further research, in the case of RA this SNP has been strongly associated with RA susceptibility by multiple studies [82,83,84,85]. Another study in a Chinese population showed that patients with PsO who carry the minor allele might have a better response in acitretin therapy [72,80,86].

In the case of rs361525, located 2KB upstream from the *TNF* locus, the A allele was found to be protective against AS and it was associated with a later age of onset as well as a lower erythrocyte sedimentation rate (ESR). Serum TNF-α levels were not significantly different and anti-TNF treatment was not influenced. However, another study suggested that patients carrying the G allele could predict a better response in etanercept therapy (an inhibitor that targets TNF) [87,88]. In the case of RA, the G allele was shown to confer risk, while patients carrying this allele also showed elevated TNF serum levels, but also had a better response to anti-TNF treatment for that reason [89]. Also, the minor allele is strongly associated with PsO type I (characterized by the early onset of symptoms) but seems to be protective of AS. Specifically, patients carrying the GA/AA genotype developed symptoms at a later age and they had a lower erythrocyte sedimentation rate (ESR); however, they gave no insight regarding serum TNF-α levels and responses to anti-TNF [88,90].

Lastly, the intronic rs1217414, located at intron 1 of the *PTPN22* locus, might affect splicing, leading to abnormal *PTPN22* expression levels. The TT genotype of this SNP has been suggested to confer risk to AS, as shown in a meta-analysis; however, another study conducted in a Han Chinese population suggested that this SNP might be protective [91,92]. This SNP has also been suggested to be protective in RA Han Chinese patients [93]. The rs1217414 in the *PTPN22* locus seems to cause splicing dysregulation by retaining additional introns after splicing (which can lead to the expression of a dysfunctional protein or the alteration of expression levels), and the T allele has been suggested to confer risk for AS. The encoded protein of this gene is thought to suppress the TCR signal transduction of T cells, and polymorphisms in this gene have been associated with many autoimmune diseases [91]. This SNP was also significantly associated with type I psoriasis (early onset), as shown in another study [94].

Beyond the genetic variants shared across AS, RA, and PsO, another set of SNPs appears to be specifically shared between AS and RA, suggesting a partially overlapping genetic architecture distinct from that of PsO, as discussed in the next section.

#### 3.2.1. The Genetic Basis Unique to Ankylosing Spondylitis and Rheumatoid Arthritis

RA and AS are considered to be two of the most common rheumatic (joint) conditions; however, there are some etiological and clinical differences between these two. The most distinct clinical difference, which was found in 1974, is that RA patients often show elevated levels of rheumatoid factors in the blood, whereas AS patients are seronegative. This has helped clinicians to better categorize AS as part of seronegative spondyloarthropathy (SpA), and to make a more accurate diagnosis, since AS was often misdiagnosed as RA. Another difference is that RA usually affects the smaller joints, such as in the hands and feet, whereas in AS the spine and especially the lower vertebrae are primarily affected [95,96]. In the case of RA many types of immune cells, such as macrophages, T and B cells, fibroblasts, chondrocytes, and dendritic cells seem to play a role, while pathologic activation of the osteoclasts, a process carried out by fibroblast-like synoviocytes (FLSs), leads to damage of the cartilage, bones, and tendons [97].

Possible SNPs that are associated with both RA and AS were gathered. In total, 22 polymorphisms were found, 4 of which are located at an exonic region, causing a missense variation, while the rest are intronic, regulating gene expression (Figure 4, Table 4). Most of the genes reported play a role in immunological processes, such as interleukin signaling, complement activation, and T-lymphocyte activation, as well as in other biological processes, including cell proliferation, differentiation, and apoptosis. Previous studies have also supported the idea that there is an interplay between both complement activation and regulation by T cells, which activates and maintains the process of inflammation in RA, confirming these findings [98]. Also, interleukin signaling has previously been studied in association with RA, as will be discussed below. Further analysis using DAVID tools and cross-validation by Reactome showed that most of the encoded proteins are located at the extracellular space and more specifically in receptor complexes, such as the *IL-23* receptor complex. Also, it was shown that most of the proteins play a role in the cytokine-mediated signaling pathway, the positive regulation of *IFN-γ* production, the cellular response to lipopolysaccharide, the positive regulation of gene expression, and the positive regulation of the Th-17-type immune response, while these are involved in molecular functions that include protein binding and, more specifically, *IL-1* and *IL-12* receptor binding.

As mentioned earlier, the *IL23*/*IL17* axis has been widely studied as it is known to be associated closely with AS and it has been used as a potential therapeutic target, like in the case of the anti-IL17 drug ‘Secukinumab’, which has been approved in the USA and by several countries in the EU [99]. Even though RA and AS include chronic inflammation mediated by the immune system and the *IL23*/*IL17* axis is thought to play a critical role in both [100], there are some differences in the therapeutic approaches used for each. In the case of RA, there are still no anti-IL17 approved drugs; however, there are novel approaches that are being used in the clinic, targeting cytokines such as *TNF*, *IL-1*, *IL-1R*, *IL6*, and *IL6R*, which have been proven to be useful [101]. Also, the *IL-12* family has been previously reported to play a role in RA by promoting T cell differentiation to Th-1, which subsequently leads to IFN-γ production, while *IL-1* is known to play a role in osteoclast differentiation, which leads to bone resorption [102,103].

In this study, many of the AS and RA common variants were found to affect interleukins. More specifically, two SNPs located at the *IL23R* locus were found, which are in non-coding regions (rs10889677 and rs1004819). In the case of rs10889677, a 3′ UTR variant was found to be associated with both AS and RA susceptibility in the general population, especially in Caucasians [104]. Furthermore, another study performed in RA Iranian patients indicated that this SNP might be associated with the overexpression of *IL23R* [105]. In the case of the intronic rs1004819, the A allele has been associated with AS susceptibility in the overall population; however, in the subgroup analysis, no significant association was shown with an Asian population [106]. It has also been associated with an increased risk of RA in Turkish and Iranian populations; however, another meta-analysis suggests otherwise, and further research is therefore needed [107,108,109].

Other SNPs located in coding regions of interleukin genes are rs3811047, rs3811058, and rs17561, affecting the genes *IL37*, *IL1F10*, and *IL-1A*, respectively. rs3811047 is located at a coding region of the *IL37* locus, causing a missense variance and is associated with AS susceptibility in the Han Chinese; however, a meta-analysis showed otherwise, meaning that more research is needed to assess its susceptibility character [110,111]. It has also been suggested to be strongly associated with more severe RA disease activity [112]. rs3811058 located at exon 3 of *IL1F10*, causing a missense variance, has been shown to be involved in AS risk; however, it was restricted to a non-AS phenotype [113]. Also, it has been strongly associated with RA and with slight differences in CRP levels [114]. The rs17561, located at exon 5 of *IL-1A*, causing a missense variance (Ser114Ala), has been shown to confer risk to both AS and RA. This SNP has been shown to influence *IL-1* expression and to be more resistant to calpain cleavage, without which *IL-1A* cannot be activated [115,116].

Other SNPs affecting genes that pertain to immunological processes include rs1800629, located 2 KB upstream of the TNF locus. Individuals carrying rs1800629 in a Bulgarian population had a lower risk of developing AS. Also, as was shown in a later study, RA and AS patients carrying the GA genotype instead of the GG genotype did not respond to anti-TNF treatment [81,117]. However, these results may vary depending on ethnicity [118]. Other SNPs also include rs3091244, a 2KB upstream variant located at the *CRP* locus, which has been associated with both AS and RA susceptibility. More specifically, it has been shown that AS patients carrying the A allele had higher *CRP* levels and that they responded better to etanercept treatment, as was shown by their ASAS20 and ASAS40 scores. For that reason, the study suggested that this SNP should be taken into consideration when assessing disease score in AS patients [119]. In the case of RA patients, the A allele contributed to higher CRP levels; however, this finding was secondary when assessing the disease score in patients whose disease had progressed [120,121]. Another intronic variant, rs11065898, located at the *SH2B3* locus, which has been positively associated with CD4+ lymphocyte counts, was suggested to be a risk factor for AS in GWASs, while only a marginal association was found between this SNP and RA in a Taiwanese population analysis [22,122].

Other SNPs that affect genes involved in other biological processes as well as other genetic loci, such as pseudogenes, include rs2283790, an intronic variant located at the *UBE2L3* gene. This SNP was associated with AS in European populations in GWASs and this was confirmed by a second analysis on a Chinese population, where a marginal association was found [22,87]. Additionally, it reached the genome-wide significance threshold in an RA study; however, there are no studies reporting the possible functional consequence that it may have [123]. Furthermore, rs13202464, which is located upstream of the *FGFR3P1* pseudogene, is a tag SNP, which strongly represents the risk effect of HLA-B*27 and has been previously shown to be a risk factor for AS in both European and Chinese populations [124]. However, it should be taken into account that this screening marker may not be suitable for every population due to differences in genomics and metagenomics [125]. This SNP has also been shown to be associated with RA in a genome-wide study [123]. Lastly, three SNPs, namely rs6759298, rs12186979, and rs2836883, which affect *RN7SL51P*, *PTGER4*, and *RPL23AP12*, respectively, have been associated with AS susceptibility in GWASs, while they were also used in an RA association study, investigating a possible connection between these SNPs and ACPA+ RA genetic risk score [22,78]. More specifically, rs6759298, an intronic variant located in a gene desert at chromosome 2p15, has been significantly associated with AS in both European and Chinese populations, as shown in GWASs [22,126]. It is suggested that this SNP might impact non-coding RNA sequence or transcription effects [127]. The variant rs12186979, located in an intronic region at *PTGER4*, as well as rs2836883 and an intergenic SNP located nearby the pseudogene *RPL23AP12* were shown to be associated with AS in European populations in GWASs, but further research is needed to assess their association as well as their functional consequences [22].

#### 3.2.2. The Genetic Basis Unique to Ankylosing Spondylitis and Psoriasis

In this study, the number of common genetic associations between AS and PsO was higher than that of AS and RA, an autoimmune disease widely thought to be closely related to AS. In the case of PsO, an excessive proliferation of epidermal cells with a simultaneous inflammation at the dermis (the middle layer of skin) leads to the creation of patches of red thick skin which causes itchiness to the patient and the characteristic lesions of PsO. It is a complex disease with no clearly defined causes, but it has been hypothesized that the amplification of this disease is T-cell-mediated, while in the pathophysiology of the disease multiple types of cells, including dendritic cells, T cells, and keratinocytes, are involved in its initiation and maintenance [128].

Based on the results, 25 variations were found to be overlapping between the two diseases, 7 of which are located at an exonic region, causing a missense variation, while the rest are intronic, regulating gene expression, modifying regulatory nucleic acid binding reactions, or dysregulating splicing (Figure 4, Table 5). Most of the genes reported play a role in immunological processes, such as trimming HLA class I-binding precursors, promulgating cytokine signals, and regulating T cell receptor signaling. This is not surprising, since it has been previously supported that professional antigen-presenting cells (APCs) play a central role in PsO pathogenesis, while activated T cells promote inflammation via cytokine secretion [129]. Other genes were also found to be involved in biological processes such as gene expression, signal transduction, amino acid metabolism, coagulation, cell differentiation, and apoptosis. Further analysis using DAVID tools and cross-validation by Reactome showed that most of the encoded proteins are located at the external side of the plasma membrane (mostly receptors), while one-fifth of them are located at the ER lumen. Additionally, it was shown that most of the proteins play a role in the tyrosine phosphorylation of the STAT protein, as well as in activation of JAK, the production of IFN-γ, and the regulation of osteoclast differentiation. Indeed, a strong association has been previously observed between psoriatic inflammation and the differentiation of osteoclasts, which can lead to PsA, a type of SpA that is similar to RA but has been linked to PsO, yet the underlying pathogenic mechanisms are not completely understood [130]. Lastly, the analysis showed that the affected proteins are involved in molecular functions of interleukin signaling and, more specifically, of *IL-6* and *IL-12*.

Both *IL-6* and *IL-12* have been previously reported to play a role in PsO. More specifically, it has been suggested that in the process of PsO pathogenesis, an initial trigger causes stress to keratinocytes, which, in response, activate plasmacytoid dendritic cells that secrete *IFN*, *TNF-a*, *IL-1β*, and *IL-6*. Subsequently, myeloid dendritic cells are activated and secrete, among others things, *IL-12*, inducing the Th-1 response, which contributes to the recurring cycle of psoriatic plaque creation [128]. Also, it has been shown that treatment that blocks the *IL-12*/*IL-23* signaling pathway seems very promising for PsO patients [131].

In this study, one SNP located in the *IL23R* locus was found at a non-coding region (rs11209032). More specifically, the minor allele seems to confer risk for both AS and PsO, as shown in GWASs [70,71,72,73]. The intergenic rs11209032 is located within an enhancer region and seems to be involved in elevated Th1 cells [72]. On the other hand, the exonic rs11209032 is a well-studied SNP that is thought to be protective of several immune-mediated diseases, among which are AS and PsO [68]. It involves a change in the negatively charged Arg to a neutral Gln in position 381, in the intra-cytoplasmic tail of the receptor, perhaps affecting interaction with *JAK2* and, therefore, reducing the signal transduction of *IL23* [65]. One other intronic variant was found within the *IL12B* locus (rs6556416), which was associated with AS and PsO in GWASs [73]. Another intronic variant located within *IL1R1* and *IL1R2* loci (rs4851529) is associated with many chronic inflammatory diseases, including AS and PsO, and seems to modulate the *IL-1* response [22,132].

Besides the involvement of interleukins in AS susceptibility, there have been many previous reports discussing the interplay between *ERAP1* and *HLA-B27* in AS susceptibility, highlighting a possible epistatic interaction, since *ERAP1* polymorphisms only affect AS risk in *HLA-B27*-positive individuals [133]. *ERAP1* is responsible for shaping the *HLA-B27* peptidome (the trimmed precursors that will bind to MHC-I molecules such as *HLA-B27*), while *HLA-B27* is responsible for presenting these peptides to T cells, determining the immunological reaction [134]. In this study, six SNPs in the *ERAP1* locus were revealed to confer risk for both AS and PsO, located at exonic regions (rs30187, rs27044, rs10050860, rs17482078, rs26653, and rs2287987) and affecting enzymatic activity (which alters trimming and therefore the *HLA-B27* peptidome) [134,135,136,137]. The variants rs30187 (K528R) and rs27044 (Q730E) seem to be protective of AS and patients who carry them express significantly lower levels of HLA class I FHCs [138]. However, two GWASs associated them with PsO susceptibility [139,140]. Likewise, the minor alleles of variants rs10050860 (D575N), rs17482078 (R725Q), rs26653 (R127P), and rs2287987 (M349V) seem to alter ERAP1 trimming and confer protection from AS and PsO. For example, the R725Q substitution of rs17482078 is thought to lead to the loss of two hydrogen bonds between R725 and D766, affecting the stability of the C-terminus of ERAP1, leading to impaired ERAP enzymatical activity [137]. Also, the variants rs26653 and rs2287987 showed the same pattern as rs30187, according to a meta-analysis between trans-ethnic populations [136]. Altogether, previous studies have confirmed that each *ERAP1* SNP contributes to AS susceptibility individually rather than by the haplotypes of SNPs [141]. Two other intronic variants were found in the *ERAP2* locus (rs2910686 and rs2248374), from which rs2910686 seems to confer risk to both AS and PsO, while the minor allele of rs2248374 seems to be protective to AS, due to leading to the complete absence of *ERAP2* expression, while it was shown to confer risk in PsO [22,132,142,143]. Lastly, it is worth noting that the association of *ERAP2* with AS appears independent of the presence of *HLA-B27*, in contrast to *ERAP1* [142].

Other intronic variants affecting proteins involved in immunological processes are the primary associated SNP at *TNFRSF1A*, rs1860545, which was found to be associated with both AS and PsO in two GWASs [22,132]. Another SNP is rs10865331, found in an intergenic region on 2p15. This region has been strongly associated with AS in Europeans in previous GWASs [23,144] and has also been found to be associated with PsO in another GWAS [145]. It has also been associated with the *BASFI* (an index used for the estimation of the disease activity of AS), and in the case of AA/AG genotypes it has been associated with a higher ESR in AS patients of a Taiwanese population [146]. Some studies suggest that this region may also affect the promoter of *COMMD1*, a gene involved in *NF-κB* signaling and located over approximately 435 kb upstream [147]. The intronic rs35164067 located in *CDC37*, which encodes a protein that promulgates cytokine signals, was also genotyped in GWASs and it was found to be protective of PsO, while on the contrary was found to confer risk to AS [22,79].

Intronic variants that affect proteins of other biological processes include rs6600247, which has been associated with both AS and PsO in GWASs, and is located in a 15 kb LD block upstream of the *RUNX3* promoter and is associated with lower CD8+ T cell counts. It has been suggested that it may disrupt a binding site of the c-MYC transcription factor, affecting the regulation of *RUNX3*, a transcription factor that also functions as a tumor suppressor [132,148]. Lastly, two significantly associated SNPs located in intergenic lncRNAs (possibly acting as transcriptional regulators) were extracted from GWASs. These are rs11624293, which is found in LINC01147, and rs11190133, found between LINC01475 and *GOT1-DT*, two ncRNAs [22,132].

### 3.3. Genetic Overlap Between AS and More Distantly Related Autoimmune Diseases—IBD, SLE, Uveitis, and MS

In addition to RA and PsO, the genetic overlap between AS and autoimmune diseases with lower association scores—namely IBD (CD and UC), SLE, uveitis, and MS—was examined and the analysis was performed in sets of three-disease combinations (Table 6, Figure 4). In all of these diseases persistent inflammation is central, each affecting a different type of tissue. IBD, encompassing CD, UC, and unclassified IBD (when patients have features that overlap CD and UC), involves chronic intestinal inflammation triggered by genetic, microbial, environmental, and immune factors. Genome-wide studies have identified over 200 susceptibility loci, from which 137 are shared between CD and UC and most are non-coding, affecting gene regulation. Both the innate and adaptive immune systems are implicated in IBD pathogenesis, with CD being associated with *Th1*/*Th17* responses, producing cytokines like *IL-17*, *IFN-γ*, and *TNF-α*, whereas ulcerative colitis features a Th2 response, with *IL-5* and *IL-13* activating B cells and NK T cells [149,150]. Although IBD primarily targets the gastrointestinal tract, it frequently leads to extraintestinal manifestations. These involve (but are not limited to) the musculoskeletal, dermatologic, hepatobiliary, and ocular systems [151]. Epidemiological studies show that clinically apparent IBD occurs in approximately 6–14% of AS patients, while microscopic gut inflammation is present in up to 60% [152]. Conversely, AS develops in up to 3% of IBD patients, particularly those with CD. Mendelian randomization and transcriptome analyses further support a causal role of IBD in promoting AS development and suggest that disease activity in IBD may influence AS progression through shared immune pathways and risk gene expression profiles [153]. Moreover, insights from genetic associations suggest the implication of the gut–joint axis, involving T cell migration and dysregulated microbiota, as an underlying factor for this bidirectional interaction [152].

SLE is a chronic autoimmune disease marked by systemic inflammation and multi-organ involvement, with a strong female predominance and distinct immunopathology compared to AS [154]. Although both conditions are associated with immune dysregulation, their co-occurrence is rare, and genetic overlap remains limited. However, recent GWASs have identified shared susceptibility loci in genes regulating immune responses, such as *TNFAIP3* and *IL23R*, suggesting that subtle common pathways may exist in immune regulation [53,155]. The immunopathogenesis of SLE involves the hyperactivation of autoreactive B cells and the production of pathogenic autoantibodies, particularly anti-dsDNA and anti-Sm, in addition to dysregulated T helper cell subsets. Key roles are played by plasmacytoid dendritic cells (pDCs), which produce large amounts of type I interferons, as well as by CD4+ T cells that provide B cell help and perpetuate inflammation. Elevated levels of cytokines such as *IFN-α*, *IL-6*, and *BAFF* (B cell activating factor) contribute to chronic immune activation and tissue damage [156]. Despite the distinct clinical presentations and immune profiles of SLE and AS, emerging evidence indicates potential overlap in cytokine-mediated signaling pathways that may support the development of shared therapeutic strategies [157].

On the other hand, autoimmune (non-infectious) uveitis is a major ocular manifestation of systemic rheumatic diseases such as RA, AS, juvenile idiopathic arthritis, and PsA, and can occur independently or alongside these conditions. Uveitis refers to inflammation primarily affecting the uveal tract with multiple clinical manifestations (acute anterior uveitis—AAU, intermediate uveitis—IU, Vogt–Koyanagi–Harada syndrome—VKH, and others), classified by type (granulomatous, non-granulomatous), anatomical location (anterior, posterior, intermediate, and panuveitis), time course, and relapse. It is thought that the abnormal immune response is mediated from T cells. Several forms of uveitis are strongly linked to specific *HLA* genes, and a well-known example is the strong link between AAU and the *HLA-B27* genes, which also explains its frequent association with AS [158,159]. Other genes, including *IL23R*, which is implicated in multiple forms of uveitis (including AAU, Behçet’s disease, and VKH), also plays a role in Th17 pathway activation, and *ERAP1*/*ERAP2*, which are involved in antigen processing, are strongly correlated with AAU and VKH, as well as the CFB, which is part of the alternative complement pathway and *STAT4*, which is associated mostly with Behçet’s disease, and serves as a crucial transcription factor in the Th1 immune response [160].

Lastly, MS is also a chronic inflammatory disease; however, it is thought to be unrelated to AS, while cases for comorbidity are rare. In this disease, activated immune cells penetrate the central nervous system and cause inflammation, which leads to myelin destruction and as a result partial, or even total, loss of cognitive and physical function. In the pathogenesis mechanism, it has been suggested that an interplay between APCs and Th cells (CD4+) initiates the disease, while *IL-4*, *IL12*, and *IL23* induce Th-1 differentiation, which, subsequently, promotes inflammation. Furthermore, TNF-α and FAS ligand (a transmembrane protein produced by lymphocytes) bind to TNF receptors on oligodendrocyte cells of the neuron, initiating apoptosis [161].

In this study, four key SNPs were identified to be overlapping between these diseases, all of which are in intronic or intergenic regions (Table 5), namely rs11465804 located at *IL23R*, rs1860545 located at *TNFRSF1A*, rs1250550 located at *ZMIZ1*, and rs1495965 located between *IL12RB2* and *DNAJB6P4*. The variants are located in genes that pertain to immunological processes, immune cell signaling, cell apoptosis, and transcription regulation (among others). More specifically, *IL23R* encodes a subunit of the receptor of IL23, which is a cytokine critical for Th17 cell development and plays a key role in multiple chronic inflammatory diseases [162,163,164,165]. Also, *ZMIZ1* acts as a modulatory regulator (can either enhance or repress transcription) of STAT signaling. The *TNFRSF1A* plays a key role in immune cell signaling, particularly in the TNF-α pathway, where it mediates inflammation, apoptosis, immune regulation, and, lastly, *IL12RB2*.

The intronic rs11465804 located at *IL23R*, with an unclear functional impact, has been previously associated with susceptibility to AS, but a reduced risk for IBD as well as a lower response to infliximab in contrast to the CC genotype for IBD [166,167]. However, in another study, it has been suggested that this SNP may confer risk to IBD in Swedish, Finnish, Hungarian, and Italian populations [168]. Moreover, this SNP has been associated with uveitis in sarcoidosis in European populations; however, this was not the case with Japanese or Czech cohorts, with the variant being monomorphic in the Japanese population [169]. On the other hand, the intronic rs1860545 in the *TNFRSF1A* gene has been associated with multiple immune-mediated diseases, including AS, CD, UC, and MS through GWASs, but without well-studied functional consequences [22,132]. This SNP is in strong linkage disequilibrium with rs1800696, which causes alternative splicing that leads to the loss of exon 6 of the protein (a transmembrane domain), which normally anchors the TNFR1 protein to the cell. As a result, a soluble form of the TNFR1 receptor is produced that acts as a soluble receptor for TNF—similarly to TNF inhibitor drugs like etanercept—and inhibits *TNF* signaling. While this may reduce inflammation and be beneficial in AS, it could have the opposite effect in MS, where *TNF* inhibition is known to worsen disease, explaining the opposite direction of genetic association [22]. The intronic rs1250550 is found in the *ZMIZ1* locus and has been previously associated with both AS and MS in GWASs. In AS the G allele is the risk variant, whereas in MS the A allele is the risk factor [22]. On the contrary, the T allele was associated with a decreased risk of developing pediatric-onset CD and UC, as well as adult-onset CD, as was confirmed by GWASs, with no subsequent studies published since 2016 [170,171]. In another study, a significant overlap of this SNP was found within active and weak promoters as well as enhancers of regions that are functionally active in B cells, suggesting that genetic regions associated with MS may induce disease via the dysregulation of B cells [172]. Lastly, the intergenic rs1495965 has been suggested as a risk factor for AS by multiple studies [70,106,173], and has also been suggested to play a role in CD, as shown in a meta-analysis [174], as well as Bechet’s uveitis; however, its functional consequences are not understood [175].

In all diseases, *IL23R* played a role in disease susceptibility—as has been extensively discussed—and most of the involved variants lie in genomic regions that pertain to immune biological processes. Notably, rs1860545 seemed to play a role in multiple immune-mediated diseases, namely PsO, CD, UC, MS, and sclerosing cholangitis, suggesting a common underlying molecular mechanism. It is important to note, however, that these findings highlight only some of the most frequently referenced genetic variants and do not impose a direct association with the disease, whereas there is still a large portion of genetic associations that remain to be studied. Therefore, more research is needed to fully understand the underlying mechanisms of these diseases.

## 4. Discussion

Currently, there is a lack of consensus on the role of specific genes in susceptibility or protection against autoimmune and autoinflammatory diseases, which arises from the complex interplay of genetic, epigenetic, and environmental factors that influence disease onset and progression. Additionally, variability in study populations, differences in genetic architectures across ethnic groups, and methodological limitations in GWASs and functional studies contribute to inconsistencies in identifying definitive causal genes. Therefore, there is a growing need for the improved characterization and documentation of rare diseases, especially those that share a common genetic background with other conditions. Such efforts can help clarify the genetic distinctions between diseases by identifying more representative and disease-specific genetic markers.

A total of 76 SNPs were identified in this study, most prominently involving genes such as ERAP1 and IL23R, which are critical in immune regulation. These variants were classified into two main groups: Class A (well-established) and Class B (emerging variants). Pathway enrichment analyses highlighted the involvement of interleukin signaling, particularly IL-10 and IL-6 pathways, confirming previous studies. Notably, 16 SNPs are located at *ERAP1*, *FCRL5*, *CAST*, *ANTXR2*, *RPL23AP12*, *MICA*, *OTULIN*, *RUNX3*, *IL1A*, *ANKH*, *STIM1*, *MICA-AS1*, and *IL33/LOC107987046*, and appear uniquely associated with AS, suggesting disease-specific genetic mechanisms. In contrast, substantial genetic overlap was observed between AS and autoimmune diseases (Supplementary Figures S2 and S3). Notably, 25 SNPs were shared with psoriasis (PsO), 22 with RA, and 5 key SNPs were found to overlap with multiple autoimmune diseases (including IBD, SLE, uveitis, and MS). Moreover, nine SNPs were common to AS, PsO, and RA, highlighting potential convergent immune mechanisms (Figure 4).

### 4.1. The AS Genetic Background in Association with Current and Potential Treatments

These findings underscore the importance of considering both disease-specific and shared genetic profiles when evaluating susceptibility and therapeutic targets. Additionally, genetic data, given the complexity of these conditions, should be interpreted in the context of clinical characteristics. In the context of treatment, as has been previously reported, *IL-17* inhibition has been proven to be effective in conditions like AS and PsA, but not in IBD, whereas targeting *IL-23* has shown potential benefits in IBD but lacks similar efficacy in AS [176]. Moreover, the genetic background of AS plays a pivotal role in shaping therapeutic strategies, particularly regarding the selection and expected efficacy of biologic agents. Tumor necrosis factor (TNF) inhibitors, including etanercept, infliximab, and adalimumab, are widely used first-line biologic therapies in AS and have demonstrated broad clinical efficacy across diverse patient populations [177,178]. While their effectiveness is not strictly dependent on *HLA-B27* status, evidence suggests that individuals who are *HLA-B27*-positive may exhibit a more favorable clinical response, possibly due to distinct immunopathogenic mechanisms or a heightened inflammatory burden associated with the *HLA-B27* genotype [179,180]. *IL-17* inhibitors, such as secukinumab and ixekizumab, represent a targeted therapeutic approach grounded in genetic insights [181]. These agents neutralize *IL-17A* and have shown substantial efficacy in patients with AS, including those who are refractory to *TNF* inhibitors. This is consistent with genetic association studies implicating the *IL-23*/*IL-17* axis—particularly polymorphisms in *IL23R* and *TYK2* in AS pathogenesis [55]. More recently, *JAK* inhibitors such as tofacitinib and upadacitinib have emerged as viable treatment options. These small molecules disrupt intracellular cytokine signaling via the *JAK-STAT* pathway, a mechanism supported by GWASs identifying variants in JAK-related genes that contribute to disease susceptibility [9].

Emerging treatment paradigms in AS increasingly reflect a shift toward precision medicine, guided by a deeper understanding of the genetic architecture of the disease. For example, therapeutic responses may be influenced by polymorphisms in *ERAP1*, an aminopeptidase involved in antigen processing that functionally interacts with *HLA-B27*. Variants in *ERAP1* may not only influence disease risk but could also serve as predictive biomarkers for responsiveness to specific immunomodulatory therapies [141]. Although still in the experimental phase, gene therapy and gene-editing technologies present theoretical opportunities to alter the expression of disease-associated alleles or to modulate immune responses at the genomic level. Despite their promise, these approaches remain limited by current technical, safety, and ethical constraints. Another novel therapeutic avenue involves the modulation of the gut microbiome, which has gained attention due to emerging evidence of gut dysbiosis in AS and its potential interaction with *HLA-B27*-mediated immune responses [182]. Genetically informed microbiome-targeted therapies—such as probiotics, fecal microbiota transplantation, or microbial metabolite modulation—may offer future adjunctive treatment options.

In our own study, several SNPs previously associated with immunoregulatory pathways also showed associations with treatment responses. For example, RA and AS patients carrying the rs1800629 GA genotype did not respond to anti-TNF. Additionally, AS patients carrying the G allele of rs361525 showed a better response to etanercept, whereas RA patients carrying the same allele showed a better response to anti-TNF. Lastly, AS patients carrying the A allele of rs3091244 showed a better response to etanercept.

### 4.2. Population-Specific Effects

Several identified SNPs exhibited population-specific associations, underscoring the role of genetic diversity in AS pathogenesis. For example, our study identified several AS-related SNPs with disease-specific effects, particularly in interleukin signaling. *IL23R* variants, such as rs11209026 (missense), have been shown to reduce *IL-17A* and *IL-22* serum levels, with protective effects in AS but increased risk for RA. Similarly, rs2201841 at *IL23R* exhibits contrasting effects, being protective in RA but increasing AS susceptibility. Additionally, the *IL12B* variant rs3212227 has been associated with AS protection, while its co-occurrence with rs17860508 amplifies risk. Moreover, rs4129267 in *IL6R* was found to confer risk for AS and predict poor response to ASAS-20 treatment in Taiwanese patients, but it reduces PsO activity. Additionally, rs30187 and rs27044 were found to be protective for AS but associated with PsO susceptibility. *ERAP2* (rs2248374) conferred protection for AS but increased PsO risk. Additionally, rs361525 in the *TNF* locus was protective for AS but associated with early-onset PsO. Lastly, rs1250550 in the *ZMIZ1* locus has been linked to both AS and MS, with the G allele conferring risk for AS whereas the A allele confers risk for MS. This SNP affects regulatory regions in the B cells, suggesting a role in disease pathogenesis via B cell dysregulation. These findings enhance the importance of genetic testing before treatment and could also aid in the future in clarifying distinctions between AS subtypes (e.g., axSpA vs. nr-axSpA), which are currently difficult to separate using clinical criteria alone.

### 4.3. Limitations of the Present Study

This study is based on the analysis of previously reported AS-related SNPs and lacks raw genome-wide data (e.g., from GWASs, WGS, or WES) to perform fine-mapping, imputation, or custom association testing; therefore, this limits the resolution and interpretability of some findings (Supplementary Table S3). As previously discussed in the literature, fine-mapping methods that include expression quantitative trait loci (eQTL) analysis as well as splicing quantitative trait loci (sQTL)—alongside long-read sequencing methods that enable the improved detection of transcript isoforms—bridge this gap by identifying which gene or isoform is affected by a given variant and in what way (cis/trans), thus providing an insight into the functional consequences of the variant [183]. Both eQTL and sQLT are important because even if a variant does not change how much of a gene is made (eQTL), it might change which isoform is made (sQTL), which can alter protein function in subtle but important ways [183]. Furthermore, this analysis can be enhanced when it takes place among different cell subpopulations, allowing for the interpretation of cell-specific effects. For example, it has been shown that RA variants are enriched specifically in Tregs and CD4+ T cells, whereas in SLE variants are mostly enriched in plasmacytoid dendritic cells and B cells—thus showcasing cellular context and possible future specific drug targets [184,185]. Moreover, it is important to note that although GWASs and literature-derived datasets provide valuable insights, there are some methodological limitations, including variability in study populations, differences in genetic architectures across ethnic groups, and limitations in functional studies that contribute to inconsistencies in identifying definitive causal genes. For example, the genetic findings were aggregated from diverse studies, many of which were conducted in European populations. Therefore, the observed associations may not be generalizable to other ethnic or geographic groups. This population bias may obscure risk loci or regulatory effects that are population-specific or result in false negatives when extrapolating to under-represented groups. Additionally, GWASs frequently identify variants in non-coding regions, the functional consequences of which remain largely unknown. Although eQTL and sQTL analyses help bridge this gap, many regulatory effects are context-dependent and dynamic, influenced by developmental stage, cellular activation state, or environmental exposure—none of which are adequately captured in static GWAS data. Furthermore, GWASs inherently struggle to account for epigenetic regulation (e.g., chromatin accessibility, histone modifications), which modulates gene expression in response to both intrinsic and extrinsic cues. Finally, gene–environment interactions, such as the role of the gut microbiota, diet, and infections, are increasingly recognized as key contributors to autoimmune disease pathogenesis. To fully understand the pathophysiology of autoimmunity, multi-omics approaches (including genomics, transcriptomics, epigenomics, and proteomics) are essential for functional validation. Additionally, longitudinal and population-diverse studies are needed to account for variability across different groups. Lastly, experimental validation is crucial, as in silico and in vitro studies can only provide a snapshot of the dynamic of in vivo environments and are often prone to technical artifacts [186,187].

### 4.4. Future Directions

To advance the understanding of AS genetics and support clinical translation, future research should leverage comprehensive genome-wide datasets combined with fine-mapping and integrative QTL analyses (eQTL and sQTL). Chromatin conformation assays and single-cell technologies will also be crucial for mapping causal variants to target genes in a cell-type- and context-specific manner, as weill integrating epigenomic datasets, including DNA methylation profiles, histone modifications, and chromatin accessibility. Beyond mapping, functional validation remains essential. In vitro assays using CRISPR perturbation, reporter systems, or cellular models can confirm regulatory variant effects, while in vivo validation in transgenic or humanized animal models can establish causal links to disease phenotypes. Multi-omics integration—including transcriptomic, epigenomic, proteomic, and metabolomic data—coupled with machine learning may enable the discovery of predictive biomarkers and therapeutic targets. In clinical practice, identifying genetic interaction patterns—such as epistatic effects, haplotype blocks within genes, or pathway-based interactions, such as for the variants involved in the *IL23R/TYK2*/*STAT3* axis, which are all involved in the *IL-23*/*Th17* inflammatory pathway—could support the development of diagnostic panels and guide personalized treatment strategies, such as stratifying patients for anti-IL17 therapy based on their genetic background [188]. Furthermore, polygenic risk scores (PRSs) combining GWAS-identified variants can enable the early identification of individuals at high risk for AS, especially when integrated with environmental or clinical data in predictive models.

Finally, expanding studies to include under-represented populations and longitudinal designs will enhance their generalizability. Ultimately, establishing multi-ethnic, multi-cohort consortia will be essential to comprehensively evaluate gene–environment–microbiome interactions and develop broadly applicable therapeutic strategies. In particular, integrating longitudinal gut microbiome profiling (rRNA sequencing, metagenomics, and metabolomics) can help elucidate how AS-associated genetic variants—such as those in *CARD9* or *IL23R* [189]—modulate microbial composition and metabolite production (e.g., short-chain fatty acids, tryptophan derivatives), which in turn influence mucosal immunity and Th17 cell expansion. Such insights could inform the development of microbiome-based biomarkers or adjuvant therapies that complement immunogenetic risk stratification.

## 5. Conclusions

This study presents a comprehensive analysis of the genetic background of AS in correlation with autoimmune diseases, forming the AS DGG. A significant effort has been made to genetically map AS through the various genetic polymorphisms that have been associated with it. The results of this study offer a holistic genetic record of AS, thus contributing to a better interpretation through the various molecular mechanisms that appear to be involved in it, with the ultimate goal of better identification and diagnosis. Additionally, this significant knowledge will have great application in clinical medicine, clinical genomics, and personalized medicine in the future.

## Figures and Tables

**Table 1:** The three classes of the identified SNPs related to Ankylosing Spondylitis.

A/A	Class	Sum
1	A—strongly associated	32
2	B—highly associated	44
3	C—associated	717
**Total**		**: 793**

**Table 2:** Disease ontology terms identified from the Class A and B SNPs of the Ankylosing Spondylitis genomic grammar.

A/A	Disease Ontology	Frequency
1	Ankylosing Spondylitis	76
2	Psoriasis	25
3	Rheumatoid arthritis	22
4	Crohn’s disease	18
5	Systemic erythematosus lupus	15
6	Ulcerative colitis	13
7	Inflammatory bowel disease	11
8	Sclerosing cholangitis	7
9	Autoimmune diseases	7
10	Uveitis	5
11	Diabetes mellitus	4
12	Multiple sclerosis	4
13	Asthma	2
14	Endometriosis	2
15	Colitis	1

**Table 3:** Ankylosing Spondylitis, psoriasis, and rheumatoid arthritis common related SNPs.

SNP ID	CHR	Change	Gene	SNP Category
rs11209026	chr1	G>A	*IL23R*	Missense variant
rs4129267	chr1	C>G/C>T	*IL6R*	Intron variant
rs35164067	chr19	G>A	*CDC37*	Intron variant
rs2201841	chr1	A>G/A>T	*IL23R*	Intron variant
rs1343151	chr1	G>A	*IL23R*	Intron variant
rs10489629	chr1	T>C	*IL23R*	Intron variant
rs3212227	chr5	A>C	*IL12B*	Three prime UTR variant
rs361525	chr6	G>A	*TNF*	2 KB upstream variant
rs1217414	chr1	G>A	*PTPN22*	Intron variant

**Table 4:** Ankylosing Spondylitis and rheumatoid arthritis common related SNPs.

SNP ID	CHR	Change	Gene	SNP Category
rs10889677	chr1	C>A	*IL23R*	Three prime UTR variant
rs1004819	chr1	G>A	*IL23R*	Intron variant
rs3091244	chr1	G>A/G>T	*CRP*	2KB upstream variant
rs13202464	chr6	A>G	*FGFR3P1*	Upstream variant
rs6759298	chr2	G>C	*RN7SL51P*	Intron variant
rs1800629	chr6	G>A	*TNF*	2KB upstream variant
rs3811047	chr2	A>G/A>T	*IL37*	Missense variant
rs11065898	chr12	C>A/C>T	*SH2B3*	Intron variant
rs12186979	chr5	A>G	*TTC33*	Intron variant
rs2836883	chr21	G>A	*RPL23AP12*	Intergenic variant
rs3811058	chr2	T>A/T>C	*IL1F10*	Missense variant
rs17561	chr2	C>A	*IL-1A*	Missense variant
rs2283790	chr22	G>A	*UBE2L3*	Intron variant

**Table 5:** Ankylosing Spondylitis and psoriasis common SNPs.

SNP ID	CHR	Change	Gene	SNP Category
rs6600247	chr1	T>A/T>C	*RUNX3*	15 KB upstream variant
rs11190133	chr10	C>T	LINC01475, *GOT1-DT*	Intergenic variant
rs1860545	chr12	G>C	*TNFRSF1A*	Intron variant
rs11624293	chr14	T>C/T>G	LINC01147	Intron variant
rs10865331	chr2	A>C/A>G	*RN7SL51P*	Intergenic variant
rs4851529	chr2	G>A/G>T	LOC107985926	2KB upstream variant
rs30187	chr5	T>C	*ERAP1*	Missense variant
rs6556416	chr5	A>C/A>G/A>T	*IL12B*	Intron variant
rs2910686	chr5	T>C	*ERAP2*	Intron variant
rs11209032	chr1	G>A	*IL23R*	Intergenic variant
rs27044	chr5	G>A/G>C	*ERAP1*	Missense variant
-||-	-||-	-||-	LOC102724748	Intron variant
rs10050860	chr5	C>T	*ERAP1*	Missense variant
rs17482078	chr5	C>G/C>T	*ERAP1*	Missense variant
-||-	-||-	-||-	LOC102724748	Intron variant
rs26653	chr5	C>A/C>G/C>T	*ERAP1*	Missense variant
rs2287987	chr5	T>A/T>C	*ERAP1*	Missense variant
rs2248374	chr5	A>G	*ERAP2*	NonCodingTransc. variant
-||-	-||-	-||-	*ERAP1*	Intron variant

**Table 6:** Key SNPs between AS and more distantly related autoimmune diseases—IBD, SLE, uveitis, and MS.

SNP ID	CHR	Change	Gene	SNP Category	Disease Association
rs11465804	chr1	T>A/T>G	*IL23R*	Intron variant	AS, CD, UC, and uveitis
rs1860545	chr12	G>A/G>C	*TNFRSF1A*	Intron variant	AS, CD, UC, and MS
rs1250550	chr10	C>A/C>G	*ZMIZ1*	Intron variant	AS, CD, and MS
rs1495965	chr1	C>A/C>T	*IL12RB2*, *DNAJB6P4*	Intergenic variant	AS, CD, and uveitis

## Data Availability

All data and materials are provided as supplementary files.

## Supplementary Materials

The following supporting information can be downloaded at: https://www.mdpi.com/article/10.3390/jcm14113677/s1, Figure S1: Overview of the PRISMA flowchart; Figure S2: Genetic overlap of AS-associated SNPs from Class A across multiple diseases; Figure S3: Genetic overlap of AS-associated SNPs from Class B across multiple diseases; Table S1: The articles that have been used in this study; Table S2: AS-related SNPs—Classes A, B, and C; Table S3: PRISMA 2020 Checklist.

## Abbreviations

AAU	Acute anterior uveitis
AS	Ankylosing Spondylitis
ASAS	Assessment of SpondyloArthritis international Society
axSpA	axial Spondyloarthritis
APCs	Antigen-presenting cells
CD	Crohn’s disease
COX	Cyclooxygenase
DGG	Disease genomic grammar
DMARDs	Disease-modifying antirheumatic drugs
ERAP1	Endoplasmic reticulum aminopeptidase 1
FLS	Fibroblast-like Synoviocytes
SLE	Systemic lupus erythematosus
SpA	Spondyloarthropathies
GWAS	Genome-wide association study
HLA	Human leucocyte antigen
ILC3s	Type 3 innate lymphoid cells
IBD	Inflammatory bowel disease
IU	Intermediate uveitis
IL	Interleukin
MHC	Major histocompatibility complex
MS	Multiple sclerosis
Nr-axSpA	Non-radiographic axial spondyloarthritis
NSAIDs	Non-steroidal anti-inflammatory drugs
PSA	Psoriatic arthritis
PsO	Psoriasis
RA	Rheumatoid arthritis
ReA	Reactive arthritis
SpA	Seronegative spondyloarthropathy
SNPs	Single-nucleotide polymorphisms
T1D	Type 1 diabetes
TNF	Tumor necrosis factor
UC	Ulcerative colitis
VKH	Vogt–Koyanagi–Harada syndrome
WES	Whole-exome sequencing
WGS	Whole-genome sequencing
