# Supplementary figures and images for: The Genetic Background of Ankylosing Spondylitis Reveals a Distinct Overlap with Autoimmune Diseases: A Systematic Review
Source: J Clin Med. 2025 May 23;14(11):3677. doi: 10.3390/jcm14113677 (PMC12155728; doi:10.3390/jcm14113677)

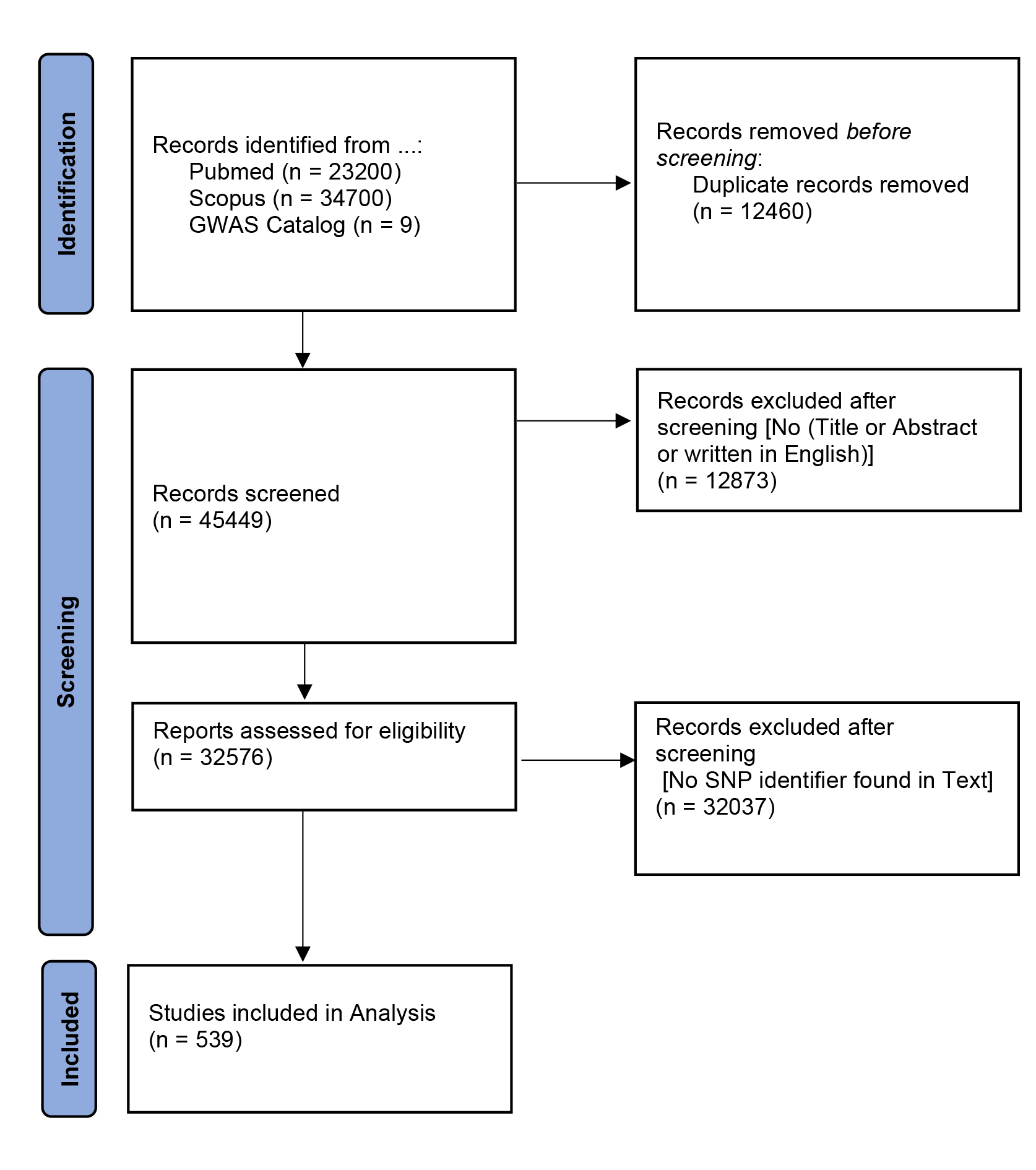

Supplement: Supplementary file 1 [file jcm-14-03677-s001.zip › Supplementary Figure_S1.jpg]

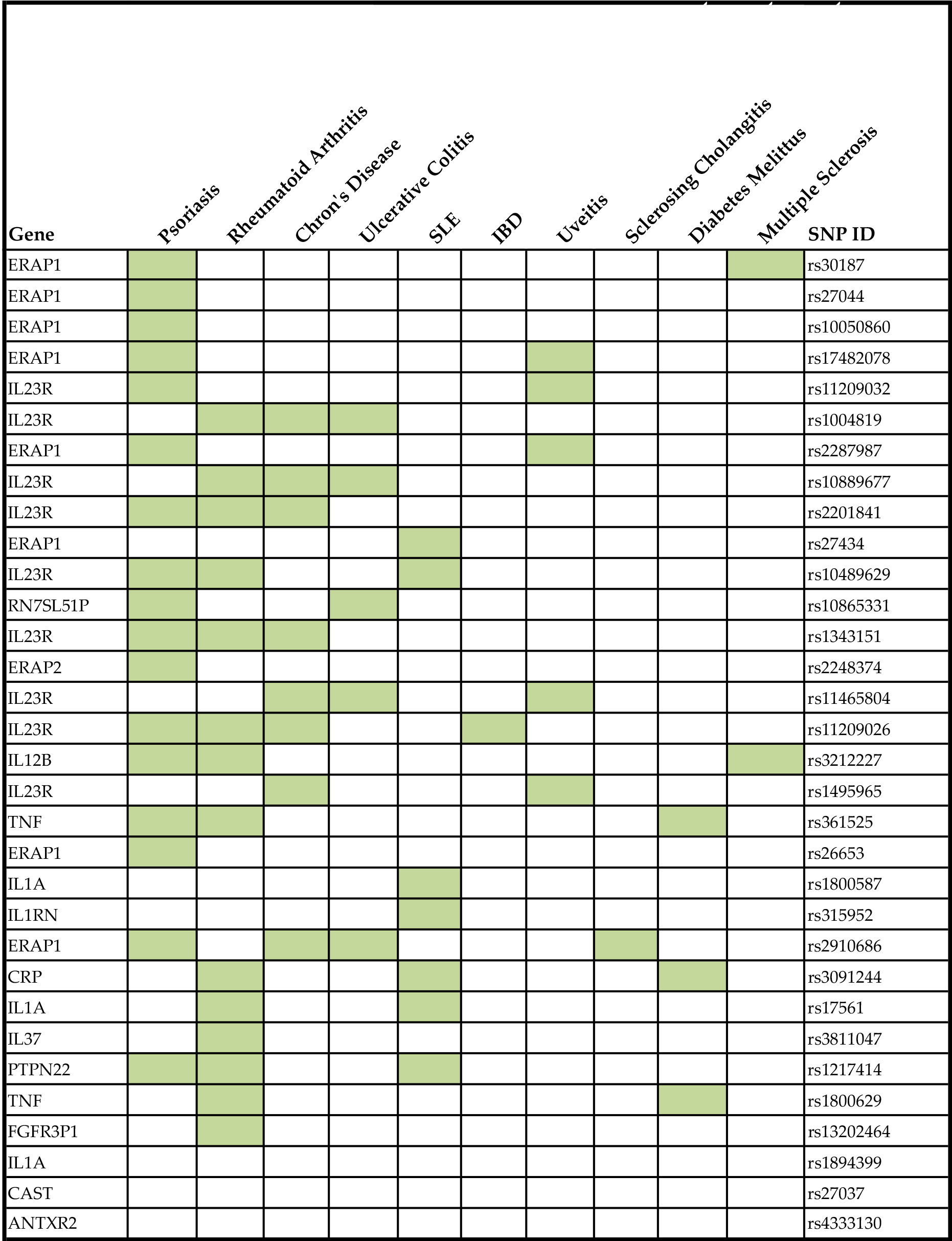

Supplement: Supplementary file 1 [file jcm-14-03677-s001.zip › Supplementary Figure_S2.jpg]

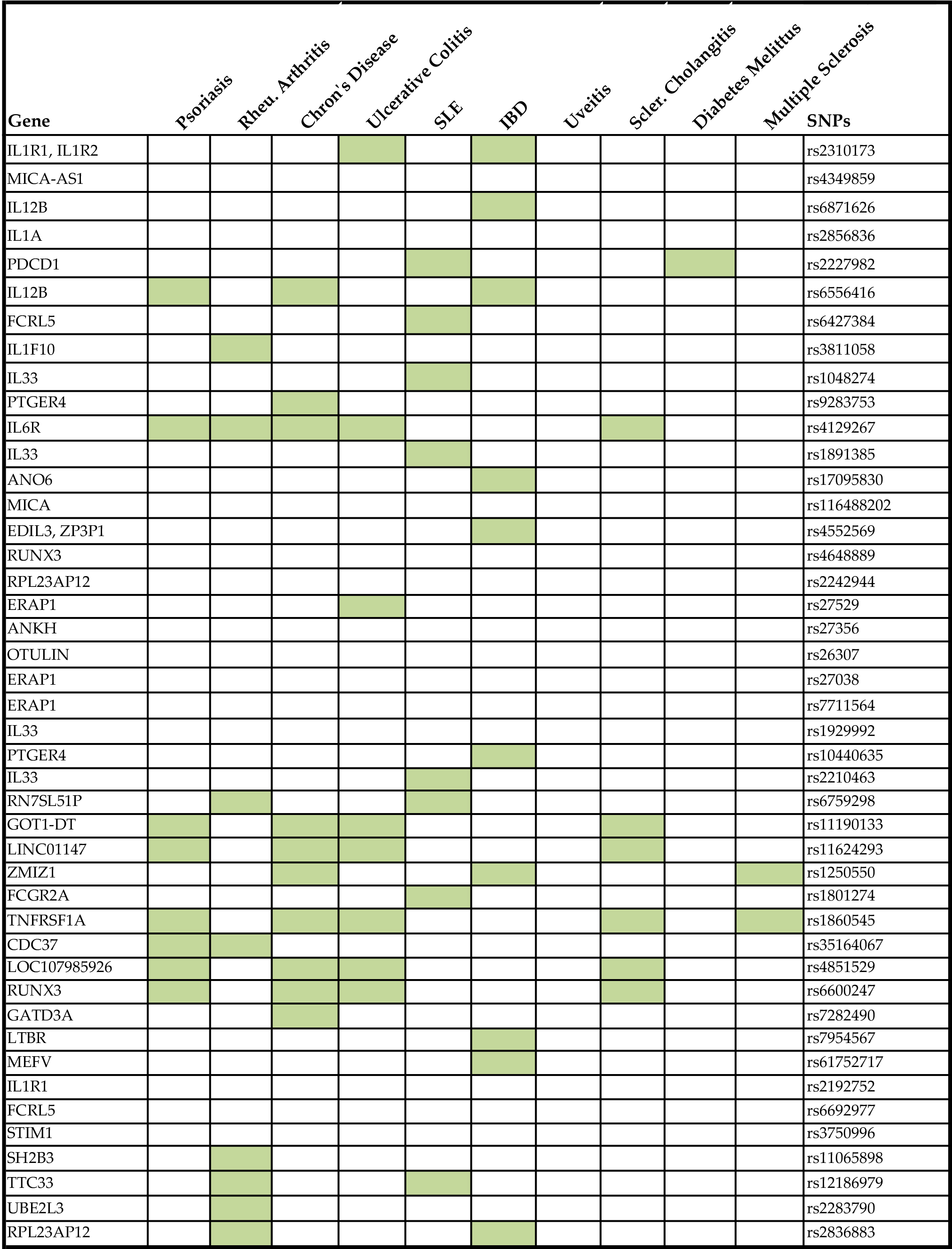

Supplement: Supplementary file 1 [file jcm-14-03677-s001.zip › Supplementary Figure_S3.jpg]
